# The Development of a Novel *Mycobacterium-Escherichia coli* Shuttle Vector System Using pMyong2, a Linear Plasmid from *Mycobacterium yongonense* DSM 45126^T^

**DOI:** 10.1371/journal.pone.0122897

**Published:** 2015-03-30

**Authors:** Hyungki Lee, Byoung-Jun Kim, Bo-Ram Kim, Yoon-Hoh Kook, Bum-Joon Kim

**Affiliations:** Department of Microbiology and Immunology, Biomedical Sciences, Liver Research Institute and Cancer Research Institute, College of Medicine, Seoul National University, Seoul, Korea; Indian Institute of Science, INDIA

## Abstract

The *Mycobacterium*-*Escherichia coli* shuttle vector system, equipped with the pAL5000 replicon, is widely used for heterologous gene expression and gene delivery in mycobacteria. Despite its extensive use, this system has certain limitations, which has led to the development of alternative mycobacterial vector systems. The present study describes the molecular structure and expression profiles of a novel 18-kb linear plasmid, pMyong2, from *Mycobacterium yongonense*. Sixteen open reading frames and a putative origin of replication were identified, and the compatibility of the pMyong2 and pAL5000 vector systems was demonstrated. In recombinant *Mycobacterium smegmatis* (rSmeg), the pMyong2 vector system showed a copy number that was approximately 37 times greater than that of pAL5000. Furthermore, pMyong2 increased the mRNA and protein expression of the human macrophage migration inhibitory factor (hMIF) over pAL5000 levels by approximately 10-fold and 50-fold, respectively, demonstrating the potential utility of the pMyong2 vector system in heterologous gene expression in mycobacteria. Successful delivery of the *EGFP* gene into mammalian cells via rSmeg carrying the pMyong2 vector system was also observed, demonstrating the feasibility of this system for DNA delivery. In conclusion, the pMyong2 vector system could be effectively used not only for the *in vivo* delivery of recombinant protein and DNA but also for mycobacterial genetic studies as an alternative or a complement to the pAL5000 vector system.

## Introduction

*Mycobacterium bovis* BCG (BCG) is a live attenuated vaccine that is used to treat tuberculosis and is currently the most frequently administered vaccine worldwide [**1**–4]. BCG represents the most stable and safe live vaccine developed; the vaccine demonstrates excellent adjuvant properties, induces long-lasting immunity, and has low production costs [5–7]. BCG also has many properties that make these bacteria attractive live vectors for the development of recombinant vaccines against other diseases. A large variety of viral, bacterial, and parasite antigens have been expressed in recombinant BCG (rBCG), many of which induce strong humoral and/or cellular immune responses following oral or parenteral immunization [8–11]. *Mycobacterium tuberculosis* proteins have been expressed in rBCG for the development of an improved tuberculosis vaccine [12]. Mycobacteria are strong adjuvants, causing them to target and persist within phagocytic monocytes. This quality makes mycobacteria attractive candidates for bacterial vectors for administering DNA vaccines, a process referred to as bactofection [6,13–15]. However, BCG vaccination does not completely clear infections in animal models [16]. *Mycobacterium smegmatis* is another effective mycobacterial vaccine vector that is non-pathogenic, grows rapidly, and can be effectively transformed *in vitro*. Currently, *M*. *smegmatis* has been employed in vaccines against various micro-pathogens [17,18]. Unlike other mycobacterial strains, such as BCG, which can survive in host cells for months by inhibiting phagosome maturation, *M*. *smegmatis* is rapidly destroyed by phagolysosomal proteases in the phagosomes of infected cells [19–21]. Nevertheless, *M*. *smegmatis* induces stronger macrophage cytokine production than other pathogenic mycobacterial species and activates dendritic cell maturation to a greater extent than BCG by up-regulating the major histocompatibility complex (MHC) class I and co-stimulatory molecules [22–24]. *M*. *smegmatis* also accesses the MHC class I pathway for the effective presentation of mycobacterial antigens [19,25].

A large number of *Mycobacterium-Escherichia coli* shuttle vectors have been developed for the transfer of foreign genes into mycobacteria [26]. These shuttle vectors are maintained in mycobacteria either episomally or through integration into the mycobacterial genome. The majority of episomal plasmids are derived from the combination of a region of the *Mycobacterium fortuitum* pAL5000 replicon with an *E*. *coli* cloning vector [27,28]. Despite high copy numbers in mycobacteria, in some cases the pAL5000-derived episomal plasmids have been associated with *in vitro* and *in vivo* instability of recombinant vaccines [2,3,29,30]. However, this reported instability may also result from promoter or protein toxicity [31,32]. Integrative vectors, derived from temperate mycobacteriophages, such as L517 or Ms6, have also been developed [33–35]. These vectors are stably integrated into the mycobacterial genome as a single copy. Thus, episomal vectors show relatively poor stability while integrative vectors are characterized by low copy number, qualities of which may compromise heterologous gene expression or bactofection in mycobacteria [13,30,36]. As a result, alternative genetic methods are required to overcome the limitations of existing mycobacterial recombination systems.

Since the first linear bacterial plasmid was identified in *Streptomyces rochei*, multiple linear, double-stranded DNA plasmids of various sizes have been isolated in Actinomycetales bacteria, including *Rhodococcus* spp. [12,33,37] and *Mycobacterium* spp. [38–40]. Among the known mycobacterial linear plasmids, the molecular details of the 23-kb pCLP from *Mycobacterium celatum* have been studied most extensively [28,41]. However, many details regarding mycobacterial linear plasmids remain unknown. In a previous study, we sequenced the complete genome of the slow-growing *Mycobacterium yongonense* DSM 45126^T^. This strain shows genetic similarity to *M*. *intracellulare*, contains 5,521,023 bp of chromosomal DNA, and harbors two additional plasmids; the first is a circular plasmid of 122,976 bp, and the second is a linear plasmid of 18,089 bp, which was designated pMyong2 [42].

Thus, the aims of the present study were two-fold. First, we aimed to elucidate the molecular characteristics of pMyong2, the linear plasmid from *Mycobacterium yongonense* DSM 45126^T^. To this end, we identified the putative open reading frames (ORFs) through an analysis of the complete sequence of pMyong2 and assessed transcriptional expression of the ORF. Second, we aimed to develop a novel pMyong2-based *Mycobacterium-E*. *coli* shuttle vector system as an alternative or complement to the conventional pAL5000-derived vector. Toward this goal, we used a bioinformatics approach to develop a novel *Mycobacterium-E*. *coli* shuttle vector system using the pMyong2 replication region. We also evaluated the pMyong2 vector system for heterologous gene expression in *M*. *smegmatis* and for potential DNA delivery into mammalian cells.

## Materials and Methods

### Bacterial strains, culture conditions, and DNA extraction

The *E*. *coli* strain DH5α (RBC Biosciences Corp., Taipei, Taiwan), purified using the PureLink HiPure Plasmid Filter Maxiprep Kit (Invitrogen, Carlsbad, CA, USA), was used as a host for all plasmid constructions. The mycobacterial strains, *M*. *yongonense* DSM 45126^T^, *M*. *smegmatis* MC^2^155 (ATCC 700084), and *M*. *bovis* BCG-Tokyo were used. The mycobacterial cultures were grown at 37°C in 7H9 broth or on 7H10 agar plates supplemented with the appropriate antibiotics. The plasmids were electroporated into competent mycobacterial cells as previously described [43,44]. Strains grown on 7H10 agar plates at 37°C were used for DNA extraction. At least one loop-full of cells was transferred to a 1.5-ml polypropylene tube containing 400 μl of TE. DNA extraction was performed as previously described [45].

### Genome sequence information from the *M*. *yongonense* DSM 45126^T^ plasmid

The whole genome sequences from *M*. *yongonense* DSM 45126^T^ have been previously described [42]. The genome sequence information for the linear plasmid pMyong2 (GenBank Accession No., JQ657806) was used to confirm the open reading frames (ORFs) in pMyong2 and to determine the mycobacterial replicon origin and the *repA*-like gene.

### RT-PCR

The *M*. *yongonense* DSM 45126^T^ strain was grown to exponential phase in 5-ml of 7H9 broth medium at 37°C. Bacterial RNA was extracted using TRIsure Reagent (Bioline, London, UK) [8], and cDNA was synthesized using the QuantiTect Reverse Transcription Kit (QIAGEN, Hilden, Germany). PCR primer pairs of approximately 19 to 25 nucleotides in length were designed to amplify the transcripts corresponding to each of the 16 ORFs, generating the expected amplicons of 134 to 665 bp in length (S1 Table). The template DNA and 20 pmol of each primer were added to a PCR tube (AccuPower PCR PreMix; Bioneer, Daejeon, Korea). The following PCR protocol was implemented: 94°C for 2 min, followed by 30 cycles of 95°C for 30 sec, 60°C for 30 sec, and 72°C for 1 min, with a final step at 72°C for 10 min and cooling to 4°C. The PCR products were examined using 1% agarose gel electrophoresis and visualized using ethidium bromide.

### Southern blot analysis

Genomic DNA was digested with *Xho*I and visualized using 1% agarose gel electrophoresis and was then transferred onto Hybond-N+ (Amersham Pharmacia biotech). The DIG High Prime DNA Labeling and Detection Starter Kit II (Roche Applied Science, Penzberg, Germany) was used for Southern blotting [46,47], and gel depurination, denaturation and neutralization steps were added [48]. The 342-bp PCR product of OEM_p200130 was used as a probe for the Southern blot analysis (S1 Table).

### Construction of a *Mycobacterium-E*. *coli* shuttle vector using a pMyong2-TOPO system

The plasmids used in the present study are listed in Table 1. The pMyong2-TOPO vector included the pMyong2 replication origin. The primer set used to PCR amplify the pMyong2 replication origin is listed in S1 Table. The PCR products were cloned using the TOPO TA Cloning Kit (Invitrogen, Carlsbad, CA, USA) according to the manufacturer’s instructions. The plasmid pMyong2-EGFP^h^ (the superscript h indicates that the plasmid contains the mycobacterial *hsp65* promoter) used for mycobacterial GFP expression contains the mycobacterial *hsp65* promoter and the *EGFP* gene. The *EGFP* gene was amplified from the pIRES2-EGFP vector (Clontech, Mountain View, CA, USA; Cat No., 6029–1), and the *hsp65* promoter gene was amplified from *M*. *bovis* BCG DNA (S1 Table). The plasmid pMyong2-EGFP^e^ (the superscript e indicates that the plasmid contains the eukaryotic CMV promoter) used for eukaryotic GFP expression contains a CMV promoter, the *EGFP* gene, and the polyA tail of pIRES2-EGFP. The *EGFP* gene was ligated into the *Nsi*I site of the pMyong2-TOPO vector. Additionally, the vectors pAL5000-EGFP-Hyg and pAL5000-mCherry were constructed for compatibility testing (Table 1). To construct the pAL5000-EGFP-Hyg vector, the pAL5000 replicon was amplified from the pSE100 vector and inserted into the pCR2.1 vector using the TA cloning method. The *EGFP* gene with the *hsp65* promoter was amplified from the pMyong2-EGFP^h^ vector and was ligated into pAL5000-TOPO using the *EcoR*V and *Not*I restriction sites. The hygromycin (Hyg) resistance gene was amplified using the Hyg primer set (S1 Table) from the pSE100 vector and was cloned into the pAL5000-EGFP^h^ vector using the *Hind*III and *BamH*I restriction sites. The pAL5000-mCherry vector contains the mCherry gene from the pmCherry-C1 vector (Clontech, Mountain View, CA, USA; Cat No., 632524) and the *hsp65* promoter in the pAL5000-TOPO vector. The mCherry gene and the *hsp65* promoter were amplified by overlapping PCR. The *hsp65* promoter region was amplified using the phsp-mCherry primer set, and the mCherry gene was amplified using the mCherry primer set (S1 Table).

**Table 1:** Vectors used in this study.

Vector	Relevant characteristics	Source
pCR2.1	TA vector	Invitrogen
pMyong2-TOPO	pCR2.1 with replicon and *rep* gene of pMyong2	This study
pMyong2-EGFP^h^	pMyong2-TOPO with *hsp65* promoter of *M*. *bovis* BCG and *EGFP* gene	This study
pMyong2-EGFP^e^	pMyong2-TOPO with cytomegalovirus (CMV) promoter and *EGFP* gene	This study
pMyong2-EGFP-Hyg	pMyong2- EGFP^h^ with hygromycin resistant gene of pSE100 (Addgene)	This study
pSE100-EGFP	pSE100 with *hsp65* promoter of *M*. *bovis* BCG and *EGFP* gene	This study
pAL5000-TOPO	pCR2.1 with pAL5000 replicon of pSE100	This study
pAL5000-EGFP-Hyg	A pMyong2 replication origin within pMyong2-EGFP-hyg is changed into a pAL5000 replication origin within pSE100	This study
pAL5000-mCherry	pAL5000-TOPO vector with mCherry gene of pmCherry-C1 vector (Clontech)	This study
pAL5000-hMIF	pAL5000-TOPO vector with *hsp65* promoter of *M*. *bovis* BCG and hMIF gene	This study
pMyong2-hMIF	pMyong2-TOPO with *hsp65* promoter of *M*. *bovis* BCG and hMIF gene	This study

### pMyong2-TOPO stability and compatibility tests

*M*. *smegmatis* carrying either the kanamycin (Km)-resistance plasmid pMyong2-TOPO or Hyg-resistance plasmid pSE100 were grown in 5 ml of 7H9 broth with no antibiotic for 24 hr at 37°C. The cultures were subsequently diluted 1:100 into 7H9 broth, and grown in fresh, antibiotic-free medium for an additional 24 hr. After each dilution, the cells were plated onto agar plates supplemented with or without Km or Hyg. The proportion of resistant cells was considered to represent the proportion of cells carrying the plasmid. The cells were also plated onto 7H10 agar plates containing 20 μg/ml of Km or 50 μg/ml of Hyg for 5 days at 37°C. For the co-transformation test, *M*. *smegmatis* was first transformed with the pAL5000-mCherry vector. Competent cells harboring the pAL5000-mCherry vector were subsequently transformed with the pMyong2-EGFP-Hyg vector. The co-transformants were selected after plating onto 7H10 agar plates containing 20 μg/ml Km and 50 μg/ml of Hyg and incubating for 5 days at 37°C. Colony PCR was used to confirm the presence of both plasmids [41,49].

### Microscopic imaging of transformed mycobacteria

For microscopic imaging of GFP expression, one visible colony of transformed *M*. *smegmatis* was obtained after incubation for 7 days at 37°C on a 7H10 agar plate. The colony was inoculated into 7H9 medium supplemented with antibiotics. One colony from the 7H10 agar plate was resuspended in 2 μl of PBS and smeared onto a slide glass. After drying, a drop of mounting buffer solution was used to raise the sample. Samples were covered with a coverslip and examined using a FV1000 laser scanning confocal microscope (Olympus).

### EGFP detection by Western blot analysis

For Western blotting, total cell lysates were prepared in RIPA lysis buffer (98% PBS, 1% Igepal CA-630, 0.5% sodium deoxycholate, 0.1% sodium dodecyl sulfate [SDS]). The protein samples were boiled for 5 min, loaded onto a 10% polyacrylamide gel, electrophoresed for 1.5 hr at 100 V, and subsequently transferred to a nitrocellulose membrane using a Wet/Tank blotting system for 1 hr at 100 V (Bio-Rad, Hercules, California, USA). The nitrocellulose membrane was blocked for 4 hr at 4°C in 5% skim milk, followed by immersion in a 1:1000 dilution of primary anti-GFP antibody (B34; Santa Cruz Biotechnology, Santa Cruz, CA, USA) in 5% skim milk overnight at 4°C. The membrane was subsequently incubated at room temperature in a 1:1000 dilution of goat anti-mouse IgG-HRP (Invitrogen, Carlsbad, CA, USA), followed by HRP-based protein detection and visualization using the ImageQuant LAS-4000 (Fuji Film).

### Determination of pMyong2-TOPO system copy number using real-time quantitative PCR

The *M*. *smegmatis* strains containing pSE100-EGFP or pMyong2-EGFP-Hyg were grown on 7H10 agar plates at 37°C until growth was clearly visible. Genomic DNA was extracted using the bead-beater method as previously described [45]. The respective genomic DNA products were used for real-time quantitative PCR (qRT-PCR). The primer pairs (S1 Table) were designed to amplify the *hsp65* gene for quantification of genomic DNA and the *EGFP* gene for quantification of the plasmid DNA. The reaction was subsequently amplified using a model LC480 qRT-PCR LightCycler system (Roche Applied Science, Penzberg, Germany). The PCR protocol was performed as follows: 94°C for 2 min, followed by 45 cycles of 95°C for 10 sec and 60°C for 30 sec, with a final melting curve. The PCR products were detected based on the crossing point (Cp) values. The raw data were analyzed using LightCycler software version 3.5. The ratio of channel 2/channel 1 signals was used to calculate the CP values.

### Expression analysis of the human macrophage migratory inhibitory factor (hMIF) gene

To compare the expression of the hMIF gene from vectors with pMyong2 and pAL5000 replication origins, the hMIF gene was cloned into both pAL5000-TOPO and pMyong2-TOPO vectors. Briefly, hMIF with the *hsp* promoter of *M*. *bovis* BCG (phsp-hMIF) was amplified using overlapping PCR with Phsp65-EcoRv-F and hmif-NotI-R primer sets (S1 Table). The pAL5000-TOPO and pMyong2-TOPO plasmids and the phsp-hMIF PCR product were digested with *EcoR*V and *Not*I restriction enzymes. Subsequently, the digested pAL5000-TOPO or pMyong2-TOPO vectors were ligated with the phsp-hMIF PCR product using the Quick Ligation Kit (NEB, Ontario, Canada) to construct pAL5000-hMIF and pMyong2-hMIF (Table 1). The constructed plasmids were transformed into the *M*. *smegmatis* MC^2^-155 strain, and the transformants were cultured in 7H9 or 7H10 medium supplemented with kanamycin, as described above. The expression levels of hMIF protein and mRNA from *M*. *smegmatis* carrying pAL5000-hMIF or pMyong2-hMIF were determined using ELISA and qRT-PCR, respectively. To conduct the ELISA analysis, the transformants were cultured in 7H9 broth (supplemented with ADC and kanamycin) and harvested, and the pellet was suspended in B-PER buffer (Thermo scientific, Rockford, IL, USA) supplemented with lysozyme (100 μg/ml), DNase (5 U/ml), and proteinase inhibitor. Subsequently, the suspensions were sonicated for 5 min (pulse: 0.3 sec, stop: 0.7 sec) on ice and centrifuged at 13,000 rpm, 4°C for 15 min. The aqueous phase was collected and analyzed using the hMIF ELISA DuoSet Kit (R&D Systems, Minneapolis, USA). The mRNA was purified from *M*. *smegmatis* transformants carrying pAL5000-hMIF and pMyong2-hMIF, and qRT-PCR was performed using primer sets for the *M*. *smegmatis* hMIF (Forward: RT-hMIF-F3, Reverse: RT- hMIF-R2) and *hsp65* (Forward: RT-hsp65-F, Reverse: RT-hsp65-R) genes (S1 Table).

### Infection of macrophages with transformed *M*. *smegmatis* and *M*. *bovis* BCG

The J774.1 cell line (American Type Culture Collection, ATCC TIB-67) was maintained at 37°C and 5% CO_2_ in Dulbecco’s modified Eagle’s medium (DMEM; Thermo Scientific, Rockford, IL, USA) supplemented with 10% (v/v) fetal bovine serum, 2 mM glutamine, and essential amino acids. Mycobacterial cultures with an optical density at 600 nm of 0.1 were used to infect macrophages. To determine the optimal inoculums for each cell line, freshly grown mycobacteria were added to the cells at a multiplicity of infection (M. O. I.) of 1, 50, or 100. The macrophages were incubated for 2 hr to allow phagocytosis of the bacteria, and the extracellular bacteria were subsequently removed by washing three times with pre-warmed, serum-free medium followed by the addition of 10 μg/ml of gentamicin. Infected J774.1 cell lines were maintained in culture for 24 hr. To detect GFP expression, infected J774.1 cells were maintained for 24 hr on a 2-chamber slide (Nunc, Rosklide, Denmark). The infected cells were washed twice with 2 ml of PBS, fixed with 4% paraformaldehyde for 20 min at room temperature, covered with a coverslip and examined using an Olympus confocal microscope (Olympus) equipped with a mercury lamp for fluorescent illumination.

To analyze GFP internalization using flow cytometry, GFP-expressing *M*. *smegmatis* or *M*. *bovis* BCG or J774 cells infected with GFP-expressing *M*. *smegmatis* or *M*. *bovis* BCG were washed four times with PBS, trypsinized, washed again with PBS, and analyzed using a FACScan or FACSCalibur cytometer and CellQuest software (Becton Dickinson). A total of 10,000 events per sample were collected. The data were further analyzed using FlowJo software (Tree Star, Inc., Ashland, OR). To analyze GFP expression in infected J774 cells, total cell lysates were prepared for Western immunoblotting using RIPA lysis buffer.

### Statistical analysis

Statistical comparisons between groups were performed using Student’s *t*-test. The level of statistical significance was set at either *P* < 0.01 (**) or 0.001 (***).

## Results

### Molecular characterization of the pMyong2 linear plasmid from *M*. *yongonense* DSM 45126^T^

The fully assembled linear 18,090-bp plasmid DNA sequence for pMyong2 was deposited in GenBank (Accession No. JQ657806). The GC content of the plasmid is 66.7%, which is typical for *Mycobacterium* spp., and the detailed plasmid information is presented in Table 2. Based on the genome sequences, a restriction map for the pMyong2 linear plasmid, containing nine restriction enzymes with a single recognition site, was constructed using the MapDraw program (DNAstar) (S1 Fig). This plasmid sequence also contains 68-bp terminal inverted repeats and covalently closed hairpin loops at both termini, suggesting that this plasmid belongs to a class of genetic elements called invertrons [40], which is similar to the linear plasmids of other Actinomycetales. We observed similarities between the terminal nucleotide sequences of pMyong2 and the sequences of known linear plasmids, including pCLP (GenBank Accession No. AF312688) from *M*. *celatum* (S2 Fig).

**Table 2:** General feature of the linear plasmid, pMyong2.

Features	pMyong2
Genome Size (base pairs)	18,089
G+C (%)	66.69
Protein-coding sequences (CDS)	25
Gene density (base pairs per gene)	723.6
Average CDS length	630.6
tRNA Number	0

**Fig 1:**
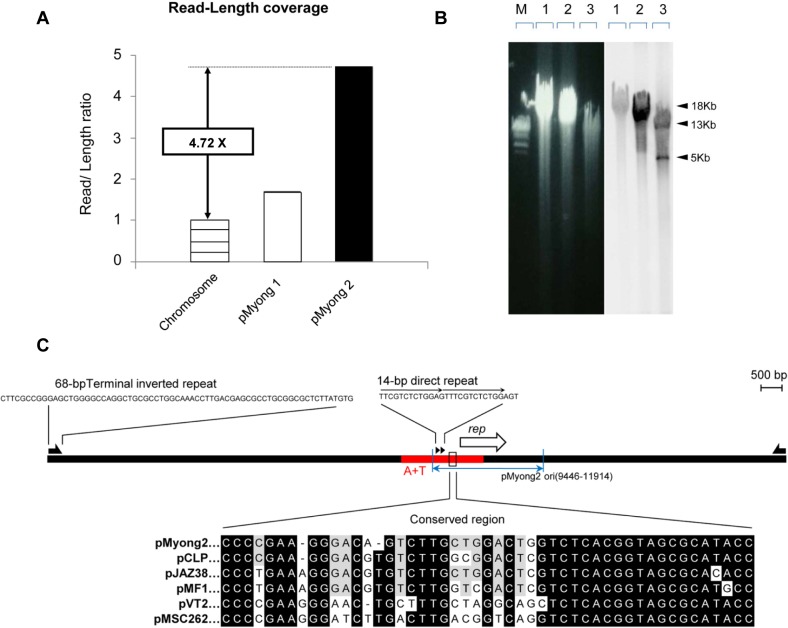
Molecular characterization of the plasmid from *M*. *yongonense* DSM 45126^T^. (A) The read-length coverage of pMyong2 was compared with the chromosome and circular pMyong1 plasmid DNA. (B) Southern blot analysis was used to assess the presence and validity of the linear plasmid, pMyong2. The left panel shows the results of gel electrophoresis, and the right panel shows the results of Southern blot analysis. The 1-kb DNA ladder (M), *M*. *intracellulare* genomic DNA (lane 1), *M*. *yongonense* genomic DNA (lane 2), and *Xho*I-digested *M*. *yongonense* genomic DNA (lane 3) are shown. (C) Comparison of the replication origin sequence in pMyong2 with those of other mycobacteria plasmids. The *rep* gene is indicated with an open arrow. The arrowheads indicate the 18-bp repeat sequences. The AT-rich segment of the replication origin region is indicated as ‘A+T’ in red. A comparison of the conserved sequences in pMyong2 with those of other mycobacterial plasmids, including pCLP (GenBank Accession No., AF312688), pJAZ38 (GenBank Accession No., MFU85216), pMF1 (GenBank Accession No., AJ238973), pVT2 (GenBank Accession No., AY0565023), and pMSC262 (GenBank Accession No., D14416), is shown. Nucleotides identical to the pMyong2 sequence are indicated with black backgrounds.

The ratio read number to the total genome size (i,e., DNA sequencing depth) was approximately 4.72 times higher for the plasmid than for the chromosome (Fig 1A), suggesting that there are approximately five copies of pMyong2 per chromosome in *M*. *yongonense*. These results are comparable to those for the pAL5000-derived plasmid [50].

In addition, the results from genome digestion with *Xho*I, using the PCR amplicon of the pMyong2 internal region as a Southern blotting probe highlighted fragments approximately 13 and 5 kb in size, supporting the linear nature of this 18-kb plasmid (Fig 1B).

Sixteen ORFs were found and seemed to be expressed in, pMyong2. Sequence homologs of the 16 ORFs were obtained from the NCBI database. A comparison of the amino acid sequences of each ORF showed percent sequence identities ranging from 31% to 85%. The pMyong2 ORFs were related to genes from rapid-growing mycobacteria [*M*. *abscessus* (4 ORFs), *M*. *rhodesiae* (1 ORF), and *M*. *smegmatis* (4 ORFs)], slow-growing mycobacteria [*M*. *canettii* (1 ORF), *M*. *celatum* (1 ORF), *M*. *tusciae* (2 ORFs), and *M*. *xenopi* (1 ORF)] and two other bacterial species (*Rhodococcus wratislaviensis* and *Gloeobacter violaceus*). Among these, the ORFs OEM_p200120 and OEM_p200150 were related to the *rep* and *parA*-like genes, respectively (Table 3).

**Table 3:** BLAST analysis of ORFs in the linear plasmid, pMyong2.

ORFs	Protein size (a.a)	Species and strains	Known or putative function	Accession No,	Identity (%)
OEM_p200020	123	*Mycobacterium rhodesiae*	Hypothetical protein	WP_005147712	38
OEM_p200040	514	*Mycobacterium smegmatis*	Transposase	YP_007277301	76
OEM_p200060	264	*Mycobacterium tusciae*	Hypothetical protein	WP_006244102	51
OEM_p200080	242	*Rhodococcus wratislaviensis*	Hypothetical protein	WP_005574670	31
OEM_p200090	806	*Mycobacterium tusciae*	Hypothetical protein	WP_006244005	61
OEM_p200100	209	*Mycobacterium abscessus*	hypothetical protein	WP_005097330	43
OEM_p200110	121	*Mycobacterium smegmatis*	hypothetical protein	YP_007277299	37
OEM_p200120	416	*Mycobacterium abscessus*	Rep-like protein	WP_005113719	43
OEM_p200130	344	*Mycobacterium abscessus*	hypothetical protein	WP_005101485	38
OEM_p200150	212	*Mycobacterium celatum*,	ParA-like protein	NP_862577	73
OEM_p200170	629	*Mycobacterium smegmatis*	hypothetical protein	YP_007277227	58
OEM_p200180	243	*Mycobacterium smegmatis*	hypothetical protein	YP_007277228	51
OEM_p200190	109	*Mycobacterium canettii*	Transcriptional modulator of MazE/toxin, MazF	YP_007267432	85
OEM_p200200	76	*Mycobacterium xenopi*	hypothetical protein	WP_003919577	75
OEM_p200220	203	*Mycobacterium abscessus*	hypothetical protein	YP_001701532	35
OEM_p200230	60	*Gloeobacter violaceus*	methionine adenosyltransferase	NP_926067	39

We also used RT-PCR to determine the transcription levels of the 16 pMyong2 ORFs in *M*. *yongonense* at the exponential stage. The *rep* (OEM_p200120) and *parA* (OEM_p200150) genes were used as transcription standards. PCR products of the expected sizes and the primers used are shown in S1 Table. Thirteen ORFs were transcribed, and 3 ORFs (OEM_p200060, p200190 and p200200) were not transcribed (S3 Fig).

Using BLAST and transcription analysis data, we identified the region containing a putative replication origin (bp: 9446 to 11914) in pMyong2. This region is located upstream of the putative *rep* gene (OEM_p200120), which is responsible for plasmid replication. The GC skew analysis showed that the putative *rep* gene belongs to a region with low GC levels, which is characteristic of mycobacteria replication origins. This region also contained a 14-bp repeat sequence (TTCGTCTCTGGAGT), another common feature of the replication origin. Furthermore, this region shared conserved signature sequences of replication origins of different types of mycobacterial plasmids (Fig 1C).

### Construction of a novel *Mycobacterium*—*E*. *coli* vector using the pMyong2 vector system

**Fig 2:**
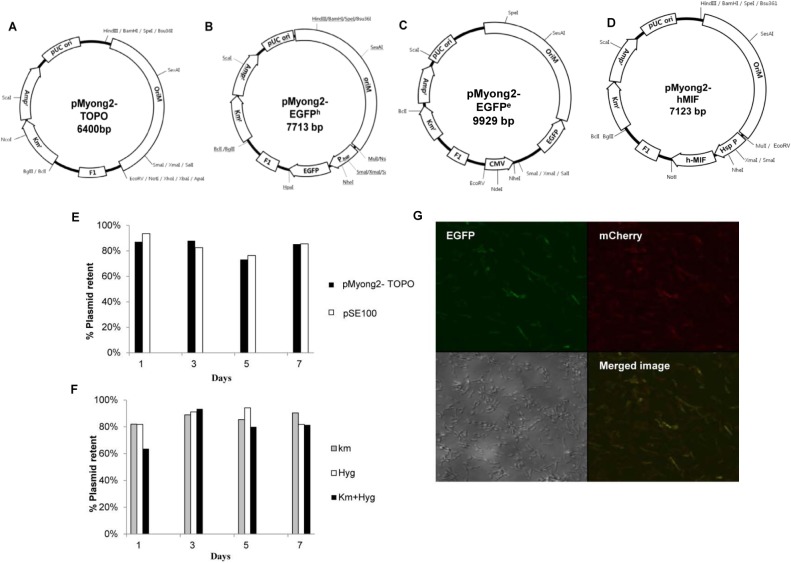
Maps of *Mycobacterium*—*E*. *coli* shuttle vectors harboring the pMyong2 vector system, and the stability and compatibility of the pMyong2-TOPO vector system. (A) Maps of the pMyong2-Topo vector, which replicates in mycobacteria and *E*. *coli*, (B) pMyong2-EGFP^h^ expressing EGFP under the mycobacterial *hsp65* promoter, (C) pMyong2-EGFP^e^ expressing EGFP under the CMV promoter, and (D) pMyong2-hMIF expressing the hMIF gene under the mycobacterial *hsp65* promoter. (E) *M*. *smegmatis*, carrying the pMyong2-TOPO (black column) or pSE100 (empty column) vectors, was plated onto 7H10 agar medium supplemented with antibiotics. (F) *M*. *smegmatis* carrying pMyong2-TOPO and pSE100 vectors was plated onto 7H10 agar medium supplemented with either or both antibiotics (gray column, kanamycin;empty column, hygromycin;black column, kanamycin and hygromycin). (G) *M*. *smegmatis* co-transformed with pAL5000-mCherry and pMyong2-EGFP-Hyg vectors was observed using confocal microscopy. Abbreviations: Amp^r^, ampicillin resistance; Km^r^, kanamycin resistance; pUC ori, bacterial origin of replication; *oriM*, mycobacterial origin of replication; CMV, cytomegalovirus promoter from pIRES2-EGFP vector; EGFP, *EGFP* gene from pIRES2-EGFP vector; P_hsp_, *hsp65* promoter from *M*. *bovis* BCG; Km, kanamycin; Hyg, hygromycin.

To develop a novel *Mycobacterium-E*. *coli* shuttle vector system using the identified replication region in pMyong2, a 2,469-bp fragment of pMyong2 (pMyong 2–2469) that included both the putative replication origin and the *rep* gene was PCR-amplified and cloned into a Km-resistant pCR2.1 TA cloning vector (Invitrogen, Carlsbad, CA, USA) (Fig 2A). This recombinant plasmid (pMyong2-TOPO) was used as a platform vector to construct a plasmid harboring the *EGFP* gene for heterologous expression (pMyong2-EGFP^h^, Fig 2B) and DNA delivery (pMyong2-EGFP^e^, Fig 2C) in mycobacteria. Additionally, to examine the heterologous expression of another gene in *M*. *smegmatis*, the *hMIF* gene was also cloned into the pMyong2-TOPO vector (pMyong2-hMIF, Fig 2D). To determine whether the pMyong2-TOPO vector was able to replicate in mycobacteria, pMyong2-TOPO was transformed into *M*. *smegmatis*, and the maintenance of pMyong2-TOPO in *M*. *smegmatis* was confirmed (S4 Fig).

### Analysis of the stability and compatibility of the pMyong2-TOPO vector

To determine the replication stability of pMyong2-TOPO in *M*. *smegmatis*, the growth of plasmid-harboring strains on plates with or without kanamycin was compared at 1, 3, 5, and 7 days after culturing. The results showed that more than 70% of transformed strains harbored plasmids even after 7 days, suggesting high stability of pMyong2-TOPO in *M*. *smegmatis* (Fig 2E). To assess the compatibility of pMyong2-TOPO with pSE100 (the hygromycin-resistant pAL5000-derived plasmid), the growth of *M*. *smegmatis* strains carrying both plasmids in 7H9 broth and on 7H10 agar plates containing both Km and Hyg antibiotics were analyzed. The strains harboring both plasmids were successfully cultivated in media containing both Km and Hyg antibiotics (> 60%) even after 7 days, suggesting that the pMyong2-TOPO vector is compatible with the pSE100 vector (Fig 2F). To confirm the compatibility of the pMyong2-derived vector system, *M*. *smegmatis* cells that had been successfully co-transformed with pAL5000-mCherry and pMyong2-EGFP^h^ (Fig 2B) were examined using confocal microscopy. The co-transformed *M*. *smegmatis* showed efficient expression of both *EGFP* and mCherry (Fig 2G). These data suggest that the pMyong2-derived plasmid may be used compatibly with the pAL5000-derived plasmid. Notably, the rSmeg strains transformed with the pMyong2 vector system (pMyong2-TOPO, pMyong2-hMIF, and pMyong2-EGFP^h^) showed a reduction in visible colony formation on 7H10 plates after transformation compared with the pAL5000 vector system (pAL5000-TOPO, pAL5000-hMIF, and pAL5000-EGFP^h^) within three days after transformation (S5 Fig). However, no significant difference in the transformation efficiency was observed between the two systems (S5 Fig).

### Comparison of the plasmid copy numbers in *M*. *smegmatis*

**Fig 3:**
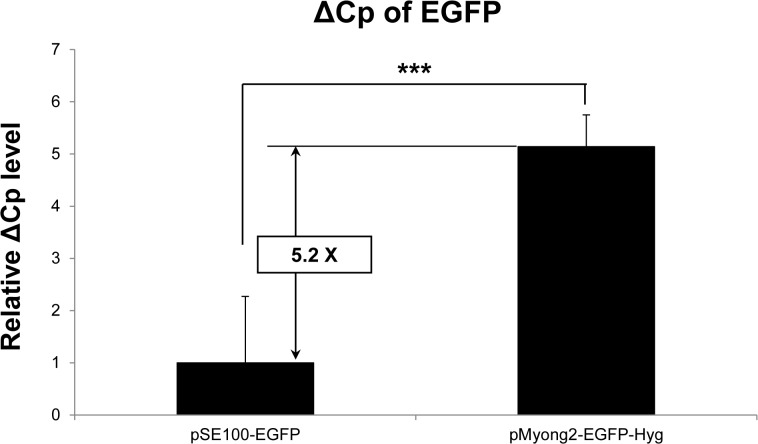
Comparison of pMyong2-EGFP-Hyg and pSE100-EGFP copy numbers. Statistical comparisons between pSE100-EGFP and pMyong2-EGFP-Hyg were analyzed using Student’s *t*-test. **, *P* < 0.01; ***, *P* < 0.001. Abbreviations: Cp, crossing point values; ΔCp, Cp value of *EGFP* normalized to that of the *hsp65* gene in transformed *M*. *smegmatis*

To compare the plasmid copy number of the pMyong2 and pAL5000 vector systems in *M*. *smegmatis*, the Cp values from *M*. *smegmatis* strains transformed with either the pMyong2-EGFP-Hyg or pSE100-EGFP plasmid were determined using RT-PCR targeting the *EGFP* gene. The Cp value for each plasmid was normalized to the Cp value of the *hsp65* gene of transformed *M*. *smegmatis* strains (ΔCp: Cp value of *EGFP* normalized to the value of *hsp65*). The ΔCp value of pMyong2-EGFP-Hyg in the transformed strain was approximately 5.2 times higher than that of pSE100-EGFP (Fig 3), suggesting that the plasmid copy number of the former might be approximately 37 times higher than that of the latter.

### Expression of the *EGFP *gene in rSmeg or rBCG using the pMyong2 vector system

**Fig 4:**
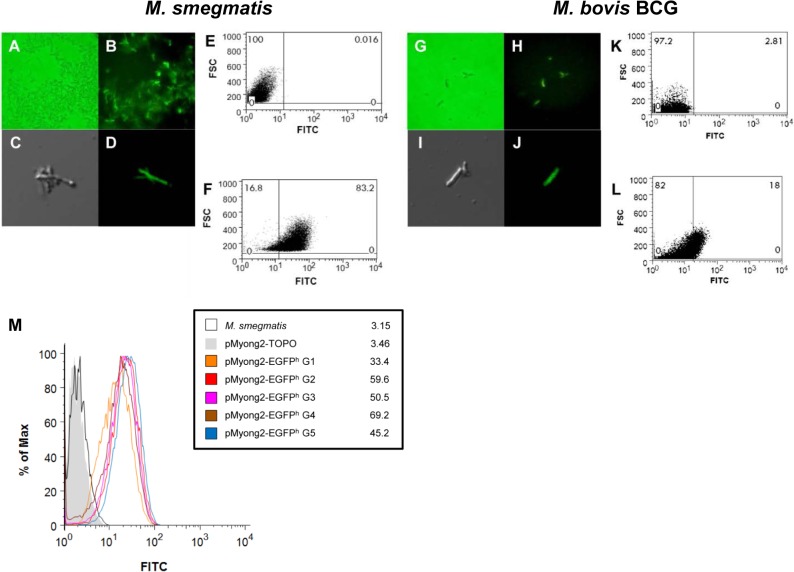
EGFP protein expression by rSmeg or rBCG harboring the pMyong2-EGFP^h^ vector. *M*. *smegmatis* transformed with the pMyong2-EGFP^h^ vector was observed using (A and B) fluorescence and (C and D) confocal microscopy. *M*. *bovis* BCG carrying the pMyong2-EGFP^h^ vector was also observed using (G and H) fluorescence and (I and J) confocal microscopy. The GFP expression levels in *M*. *smegmatis* and *M*. *bovis* BCG, transformed with (E and K) pMyong2-TOPO or (F and L) pMyong2-EGFP^h^, were detected using flow cytometry. (M) GFP expression levels in each generation of rSmeg transformed with pMyong2-EGFP^h^ were analyzed using flow cytometry. Abbreviations: G1, 1^st^ generation; G2, 2^nd^ generation; G3, 3^rd^ generation; G4, 4^th^ generation; G5, 5^th^ generation. Each generation time was 3 days.

**Fig 5:**
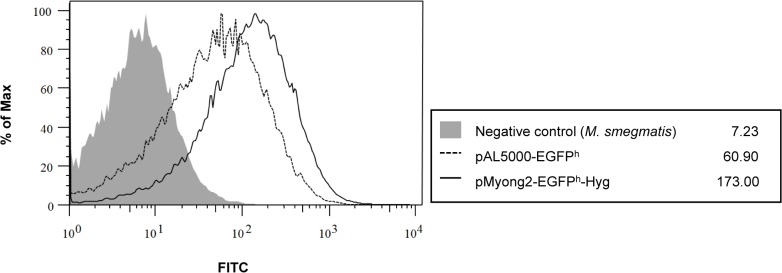
FACS analysis for comparison of GFP expression in pAL5000 and pMyong2-derived vector systems. *M*. *smegmatis* strains transformed with pMyong2-EGFP^h^ or pAL5000-EGFP^h^ were cultured and analyzed using flow cytometry to compare the GFP expression levels. gray space, Negative control; dotted lined space, *M*. *smegmatis* harboring with pAL5000-EGFP^h^ vector; black lined space,*M*. *smegmatis* harboring the pMyong2-EGFP^h^-Hyg vector.

To determine whether the pMyong2-TOPO shuttle vector system can be used for heterologous expression in *M*. *smegmatis* and *M*. *bovis* BCG, we constructed pMyong2-EGFP^h^, a pMyong2-TOPO vector expressing the *EGFP* gene under an *hsp65* promoter from *M*. *bovis* BCG (Table 2 and Fig 2B). The expression of the reporter gene in rSmeg (Fig 4A–4F) and rBCG (Fig 4G–4L) was verified using fluorescence (Fig 4A and 4B; *M*. *bovis* BCG: Fig 4G and 4H), confocal microscopy (*M*. *smegmatis*: Fig 4C and 4D; *M*. *bovis* BCG: Fig 4I and 4J), flow cytometry (*M*. *smegmatis*: Fig 4E and 4F; *M*. *bovis* BCG: Fig 4K and 4L), and Western blotting (S6 Fig). To analyze the *EGFP* gene expression levels using flow cytometry, rSmeg and rBCG, transformed with the pMyong2-TOPO vector, were used as controls (Fig 4E and 4K, respectively). Furthermore, a 15% reduction in EGFP expression was observed after the fifth-generation passage of rSmeg cultured without antibiotics compared with the first-generation passage of rSmeg, suggesting that the pMyong2-TOPO vector system is suitable for heterologous expression in mycobacterial species (Fig 4M). The GFP expression levels in rSmeg were compared between the pAL5000 and pMyong2 vector systems using flow cytometry analysis. The results showed that the level of GFP expression in the pMyong2 vector system was approximately three-fold higher than that in the pAL5000 vector system (173 vs. 61) (Fig 5).

### Expression of hMIF in rSmeg using the pMyong2 vector system

**Fig 6:**
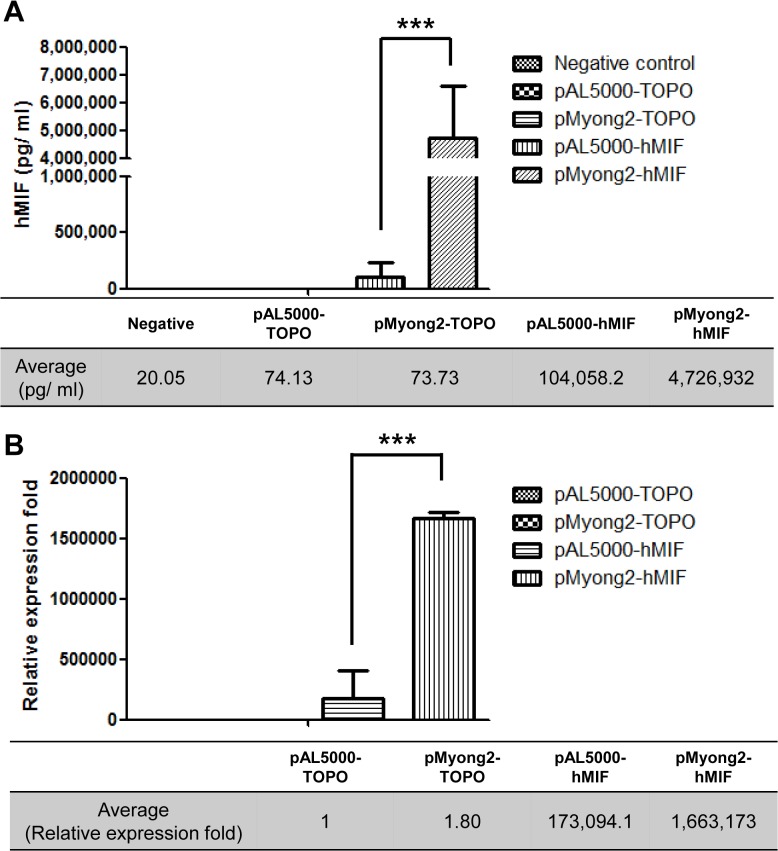
Comparison of hMIF gene expression between the pMyong2-hMIF and pAL5000-hMIF vectors. The protein and mRNA levels of the hMIF gene in rSmeg harboring pMyong2-hMIF and pAL5000-hMIF were analyzed using (A) ELISA and (B) real-time PCR, respectively. The tables show the average hMIF concentrations determined using ELISA and the relative mRNA expression levels using real-time PCR.

We used the hMIF gene to further assess heterologous protein expression in the pMyong2 vector system because hMIF is an essential proinflammatory cytokine involved in innate immunity, antimicrobial defense and the stress response [51]. To compare hMIF expression levels between the pAL5000 and pMyong2 vector systems, pAL5000-hMIF and pMyong2-hMIF vectors expressing hMIF under the same mycobacterial *hsp65* promoter were constructed (Table 3 and Fig 2D). The hMIF expression levels were evaluated at the mRNA and protein levels using real-time PCR and ELISA, respectively, from three independent transformed colonies. The ELISA results showed that rSmeg carrying pMyong2-hMIF produced approximately 47-fold higher levels of MIF (approximately 4.7 μg/ml) than rSmeg carrying pAL5000-hMIF (approximately 0.1 μg/ml) (Fig 6A). Additionally, to determine the levels of hMIF mRNA expression using real-time PCR, the ΔCp values were analyzed (ΔCp = Cp value of *hsp65* mRNA—Cp value of hMIF mRNA). The pMyong2-hMIF mRNA expression level was approximately 10 times higher than that of pAL5000-hMIF (Fig 6B). These results show that the pMyong2 vector system has an advantage over the pAL5000 vector system in the expression of heterologous genes in *M*. *smegmatis*.

### Delivery of recombinant EGFP proteins into a macrophage cell line via rSmeg or rBCG using the pMyong2-EGFP^h^ shuttle vector

**Fig 7:**
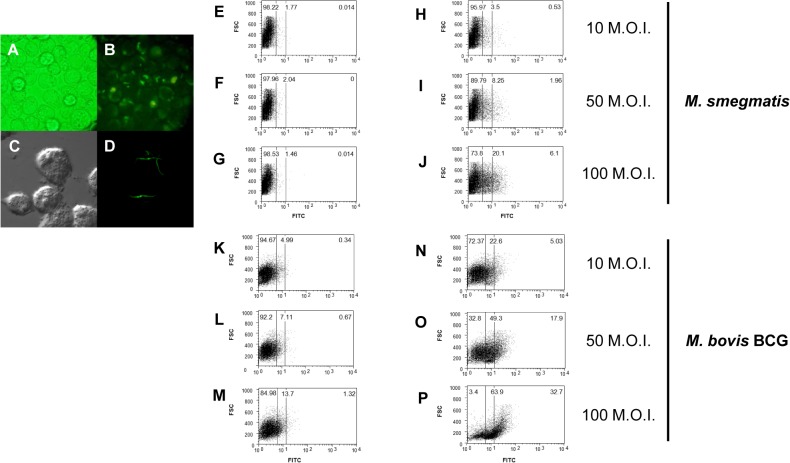
Delivery of EGFP into the macrophage cell line J774A.1 through rSmeg or rBCG carrying the pMyong2-EGFP^h^ shuttle vector. GFP expression in rSmeg harboring the pMyong2-EGFP^h^ vector was observed using (A and B) fluorescence and (C and D) confocal microscopy. A FACS analysis of the GFP expression of rSmeg harboring (E, F, and G) pMyong2-TOPO and (H, I, and J) pMyong2-EGFP^h^ at 10, 50, and 100 M. O. I., respectively. In addition, GFP expression in *M*. *bovis* BCG harboring (K, L and M) pMyong2-TOPO or (N, O, and P) pMyong2-EGFP^e^ was analyzed at 10, 50, and 100 M.O.I., respectively.

To determine whether the pMyong2 vector system could be used for the delivery of recombinant protein into macrophage cells, J774.1 cell lines were infected with rSmeg or rBCG harboring pMyong2-EGFP^h^. Twenty-four hours after infection, one to five internalized rSmeg were observed through fluorescence (Fig 7A and 7B) or confocal microscopy (Fig 7C and 7D). The FACS analysis of J774.1 cells infected with rSmeg harboring pMyong2-EGFP^h^ showed a correlation with the increase of M.O.I. (10, 50, and 100) and the EGFP expression level (Fig 7H–7J); however, rSmeg carrying the pMyong2-TOPO vector showed no changes (Fig 7E–7G). For rBCG harboring pMyong2-EGFP^h^, EGFP expression in infected J774.1 cells was similar to that of rSmeg (Fig 7K–7P). These data demonstrated the usefulness of the pMyong2 vector system for the delivery of recombinant proteins into mammalian cells.

### Delivery of recombinant *EGFP* DNAs into a macrophage cell line via rSmeg using the pMyong2-EGFP^e^ shuttle vector

**Fig 8:**
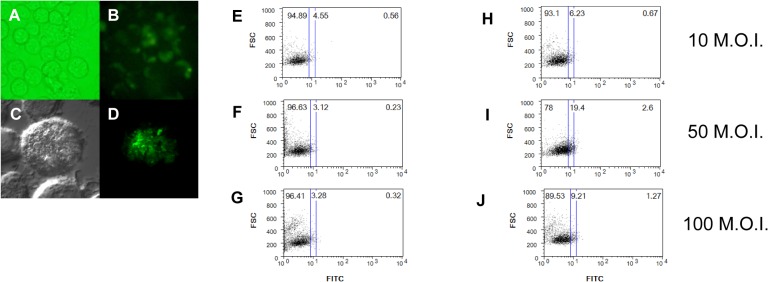
Delivery of recombinant *EGFP* DNA into the macrophage cell line, J774A.1, via rSmeg using the pMyong2-EGFP^e^ shuttle vector. GFP expression in rSmeg harboring the pMyong2-EGFP^e^ vector was observed using (A and B) fluorescence and (C and D) confocal microscopy. A FACS analysis of the GFP expression in *M*. *smegmatis* harboring (E, F, and G) pMyong2-TOPO and (H, I, and J) pMyong2-EGFP^e^ at 10, 50, and 100 M.O.I., respectively.

To evaluate the usefulness of the pMyong2 vector system for DNA delivery into mammalian cells, the mycobacterial reporter plasmid, pMyong2-EGFP^e^ (Fig 2C) was constructed and transformed into *M*. *smegmatis*. After the infection of rSmeg harboring pMyong2-EGFP^e^, the GFP expression levels were detected using fluorescence or confocal microscopy at 24 hr after infection. Interestingly, there was a striking difference in the microscopy results for infections of rSmeg with pMyong2-EGFP^h^ and pMyong2-EGFP^e^. EGFP expression was limited to bacteria infected with rSmeg harboring pMyong2-EGFP^h^ (Fig 7A–7D). However, in the case of rSmeg harboring pMyong2-EGFP^e^, EGFP expression was detected in the cytosol of macrophages after infection (Fig 8A–8D). These results suggest that the EGFP may escape to the cytosol from dead bacteria in phagocytic vacuoles via an unknown mechanism. FACS analysis also confirmed the expression of the *EGFP* gene after plasmid delivery of rSmeg using pMyong2-EGFP^e^. Similar to rSmeg harboring pMyong2-EGFP^h^, an increase in the M.O.I. also correlated with an increase in EGFP expression in infected rSmeg with pMyong2-EGFP^e^ (Fig 8E–8J).

## Discussion

The complete sequence of a novel linear plasmid, pMyong2, from *M*. *yongonense* showed at least 16 putative ORFs (Table 2). Of these, 11 ORFs were matched with hypothetical proteins (Table 3), suggesting that some of the ORFs with unknown functions might be responsible for several biological functions, including linear plasmid maintenance. The two ORFs *repA* and *parA*, which are responsible for basic functions in the plasmid, showed the closest homology to those of *M*. *abscessus* and *M*. *celatum*, suggesting a common evolutionary ancestry between two different types of mycobacterial linear plasmids (Table 3). Transposase, encoded by OEM_p200040, might have been initially introduced into the *Mycobacterium* spp., the closest match to *Streptomyces* sp. (53% similarity), suggesting that this ORF may have been transferred from another Actinomycetales member to the linear plasmid pMyong2. Given the active transcription of transposase in *M*. *yongonense*, the gene has the potential to function as a mobile element. Thus, it is likely that the OEM_p200040 product promotes gene transfer between mycobacteria (Table 3 and S3 Fig).

Our data show that the read-length coverage in the pMyong2 plasmid was 4.72 times higher than that of the chromosome, suggesting the presence of approximately five copies of the pMyong2 plasmid per chromosome, comparable to the copy numbers of pAL5000 (Fig 1A). Notably, the pMyong2 plasmid naturally shows a relatively high copy number in the slow-growing mycobacteria, *M*. *yongonense*, suggesting the potential of the pMyong2 system for the stable expression of heterologous antigens in slow-growing Mycobacteria, such as *M*. *bovis* BCG, *M*. *tuberculosis*, and *M*. *avium* complex strains. Indeed, successful EGFP expression in rBCG harboring pMyong2- EGFP^h^ supports this hypothesis (Fig 4G–4L).

The compatibility of our pMyong2-TOPO system with the pAL5000-derived plasmid pSE100 was established (Fig 2E–2G). The compatibility suggests the potential for simultaneous use of both plasmid systems encoding different genes in a mycobacterial host, which may facilitate broader applications of mycobacterial genetic manipulation beyond those offered by the pAL5000-derived plasmid alone. The primary limitation of the pAL5000-derived plasmid is the unstable expression of heterologous antigens. However, the pMyong2-TOPO system showed stable EGFP expression in *M*. *smegmatis*, even after five generations of recombinant strains (Fig 4M). However, it is not certain that this system will enable stable expression of all proteins in mycobacteria. This issue should be addressed in a future study.

MIF was one of the first cytokine described [52]. MIF demonstrates an immune modulatory function [51]; thus, recombinant mycobacteria producing MIF might be effectively used for several immunotherapeutic purposes, such as anti-cancer therapy or potentiating vaccines. Furthermore, the eukaryotic MIF is reported to have common tautomerase activity with the bacterial tautomerase [53]. Thus, we selected hMIF for the evaluation of heterologous protein expression in rSmeg using the pMyong2-TOPO system. Notably, rSmeg carrying the pMyong2 vector system produced approximately 50 times higher MIF protein than rSmeg carrying the pAL5000 system, suggesting the feasibility of the pMyong2 vector system for heterologous protein expression in *M*. *smegmatis* (Fig 6). The plasmid copy number of the pMyong2 vector system was approximately 37 times higher than that of the pAL5000-vector system, which also supports the potential utility of this system. To our knowledge, the present study is the first to demonstrate recombinant mycobacteria expressing hMIF.

The infection of phagocytes by rSmeg harbouring pMyong2-EGFP^h^ showed that the *EGFP* gene expression in rSmeg using the pMyong2 plasmid system could be successfully maintained in host phagocytes, strongly supporting the usefulness of this system for the development of vaccines using recombinant mycobacteria (Fig 7A–7J).

Although attenuated strains of *M*. *smegmatis* and *M*. *bovis* BCG, expressing heterologous antigens, are promising vaccine vectors, the efficacy of these bacteria is limited by reduced expression, the incomplete processing of full-length recombinant polypeptides within the bacteria, and the failure to engender strong immune responses to non-secreted recombinant antigens [13,54,55]. In contrast, an attenuated bacterial vector for the delivery of a DNA vaccine into mammalian cells has the distinct advantage of ensuring precise endogenous expression, the presentation of recombinant polypeptides to CD8 and CD4 T cells, and proper post-translational modifications, including glycosylation, and thereby facilitating a robust antigen-specific immune response [13].

*M*. *smegmatis* is a promising candidate vector for DNA vaccine delivery [13]. It has been previously reported that *M*. *smegmatis* mediates plasmid delivery and subsequent transgene expression despite rapid clearance in mice [36]. When J774.1 cells were infected with rSmeg harboring pMyong2-EGFP^e^, the levels of EGFP expression were lower than those of infected rSmeg harboring pMyong2-EGFP^h^ (Figs 7H–7J and 8H–8J). These results likely reflect limitations in the use of mycobacteria as vectors for DNA plasmid transfer, including the exclusive residence of these microbes in the vacuoles of infected antigen-presenting cells. For the efficient expression of the pMyong2-EGFP^e^ vector system, the plasmid must escape the vacuole and directly enter the host cell cytoplasm.

In the present study, we analyzed the complete genome sequences and elucidated the molecular details of pMyong2, a linear plasmid from *M*. *yongonense* related to *M*. *intracellulare*. In addition, we developed a new *Mycobacterium-E*. *coli* shuttle vector system using the mycobacterial replicon pMyong2 that is compatible with the pAL5000-derived vector. Furthermore, the infection into mammalian cells with *EGFP*-encoding rSmeg demonstrated the feasibility of this system for bactofection and heterologous gene expression in mycobacteria. Future studies should address the role of pMyong2 in the pathogenesis or metabolism of *M*. *yongonense*. Furthermore, the usefulness of the pMyong2 system for the development of recombinant mycobacteria for vaccination should be evaluated with several viral or mycobacterial antigens.

In conclusion, the pMyong2 vector system could be effectively used not only for the *in vivo* delivery of recombinant protein and DNA but also for mycobacterial genetic studies as an alternative or a complement to the pAL5000 vector system.

## Supporting Information

S1 Fig Restriction enzyme map of the linear pMyong2 plasmid. (PDF)

S2 Fig Short terminal inverted repeats in, pMyong2. (A) Predicted secondary structure of pMyong2 termini showing three hairpin structures. (B) The linear palindromic sequence is shown and the stem loop structures are indicated with different colors in panel A. (C) Alignment of the terminal sequences between pMyong2 and pCLP of *M*. *celatum*.
(PDF)

S3 Fig RT-PCR results for the transcription of 16 ORFs in pMyong 2. (A) ORF organization in the linear plasmid, pMyong2, indicated as arrows. The amplification of the ORFs is indicated as positive (+) or negative (-). (B) The RT-PCR results of 16 ORFs in the linear plasmid, pMyong 2. M, DNA ladder; 1, OEM_p200020; 2, OEM_p200040; 3, OEM_p200060; 4, OEM_p200080; 5, OEM_p200090; 6, OEM_p200100; 7, OEM_p200110; 8, OEM_p200120; 9, OEM_p200130; 10, OEM_p200150; 11, OEM_p200170; 12, OEM_p200180; 13, OEM_p200190; 14, OEM_p200200; 15, OEM_p200220; 16, OEM_p200230.
(PDF)

S4 Fig Growth rate of rSmeg transformed with *Mycobacterium-E*. *coli* shuttle vector, pMyong2-TOPO. Growth rate of *M*. *smegmatis* carrying pMyong2-TOPO (black and black dotted line), pSE100 (gray dotted line) and no-plasmid (gray line). Plasmid carrying strains were grown in the (A) absence or (B, hygromycin; C, kanamycin) presence of antibiotics, and the OD at 600 nm was determined at after 1, 3, 5 and 7 days.
(PDF)

S5 Fig Growth retardation due to the pMyong2 vector system. Comparison of the phenotypic differences between rSmeg carrying the pMyong2 or pAL5000 vector system. Three days after transformation, the colonies were (A) counted, and (B) the colony sizes were examined. 1, *M*. *smegmatis* pMyong2-TOPO; 2, *M*. *smegmatis* pMyong2-hmif; 3, *M*. *smegmatis* pMyong2-EGFP^h^; 4, *M*. *smegmatis* pAL5000-TOPO; 5, *M*. *smegmatis* pAL5000-hmif; 6, *M*. *smegmatis* pAL5000-EGFP^h^
(PDF)

S6 Fig Confirmation of GFP expression in rSmeg by Western blotting. Protein extracted from *M*. *smegmatis* (lane 1), rSmeg clones carrying pMyong2-EGFP^h^ for 10 generations (lane 2, 1^st^ generation; lane 3, 2^nd^ generation; lane 4, 4^th^ generation; lane 5, 6^th^ generation; lane 6, 8^th^ generation; lane 7, 10^th^ generation), and GFP protein (lane 8) as a positive control. Each generation time was three days.
(PDF)

S1 Table Primers used in this study. (DOC)
