# Supplementary figures and images for: The Development of a Novel Mycobacterium-Escherichia coli Shuttle Vector System Using pMyong2, a Linear Plasmid from Mycobacterium yongonense DSM 45126T
Source: PLoS One. 2015 Mar 30;10(3):e0122897. doi: 10.1371/journal.pone.0122897 (PMC4378964; doi:10.1371/journal.pone.0122897)

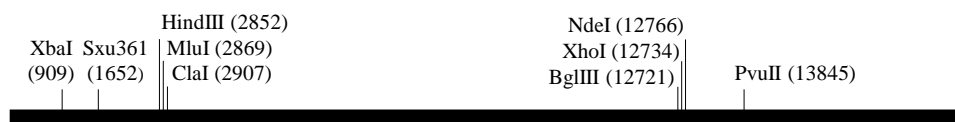

Supplement: S1 Fig — (PDF) [file pone.0122897.s001.pdf]

**A**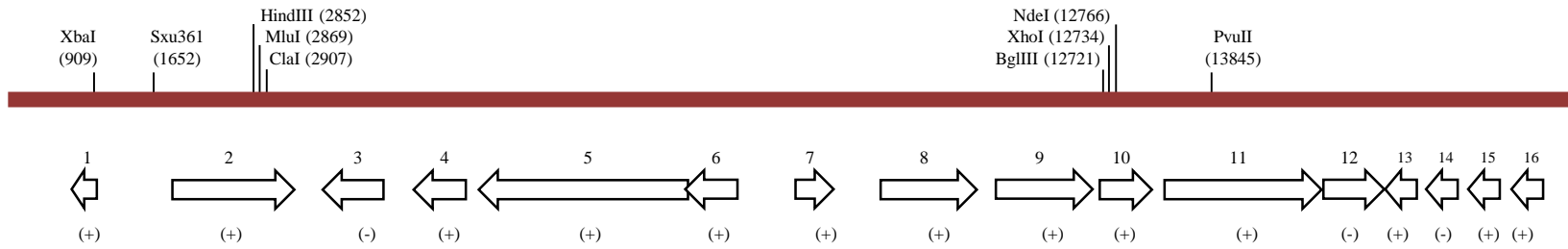**B**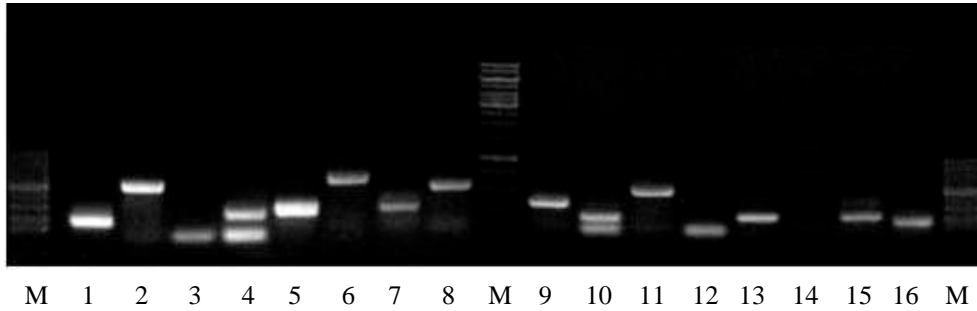

Supplement: S3 Fig — (A) ORF organization in the linear plasmid, pMyong2, indicated as arrows. The amplification of the ORFs is indicated as positive (+) or negative (-). (B) The RT-PCR results of 16 ORFs in the linear plasmid, pMyong 2. M, DNA ladder; 1, OEM_p200020; 2, OEM_p200040; 3, OEM_p200060; 4, OEM_p200080; 5, OEM_p200090; 6, OEM_p200100; 7, OEM_p200110; 8, OEM_p200120; 9, OEM_p200130; 10, OEM_p200150; 11, OEM_p200170; 12, OEM_p200180; 13, OEM_p200190; 14, OEM_p200200; 15, OEM_p200220; 16, OEM_p200230. (PDF) [file pone.0122897.s003.pdf]

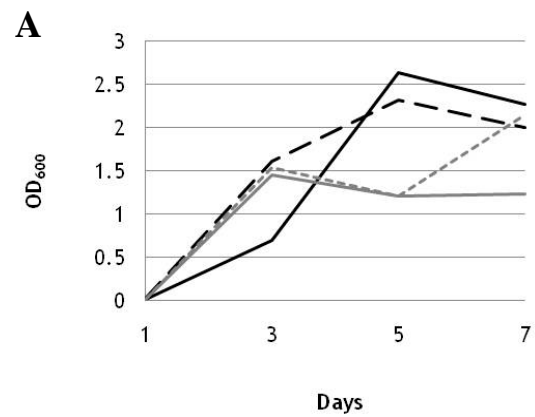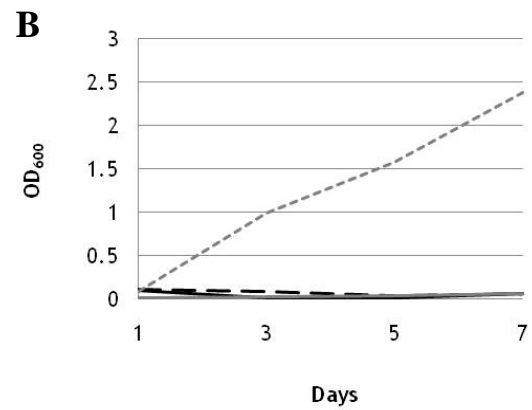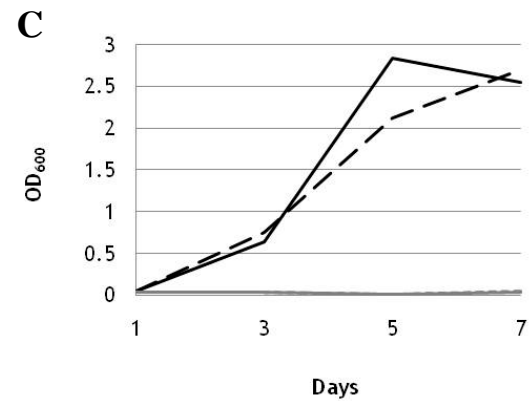

Supplement: S4 Fig — Growth rate of M. smegmatis carrying pMyong2-TOPO (black and black dotted line), pSE100 (gray dotted line) and no-plasmid (gray line). Plasmid carrying strains were grown in the (A) absence or (B, hygromycin; C, kanamycin) presence of antibiotics, and the OD at 600 nm was determined at after 1, 3, 5 and 7 days. (PDF) [file pone.0122897.s004.pdf]

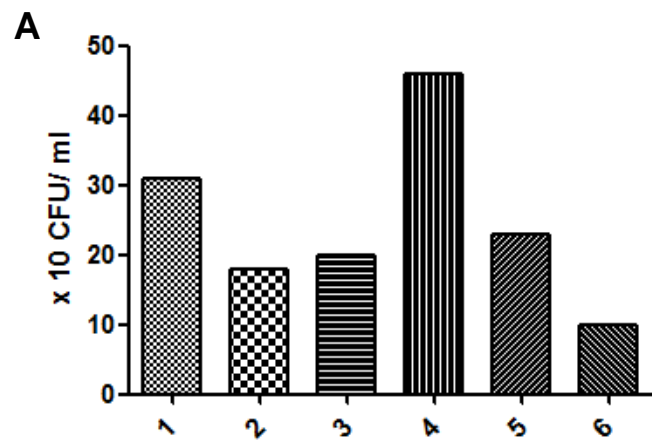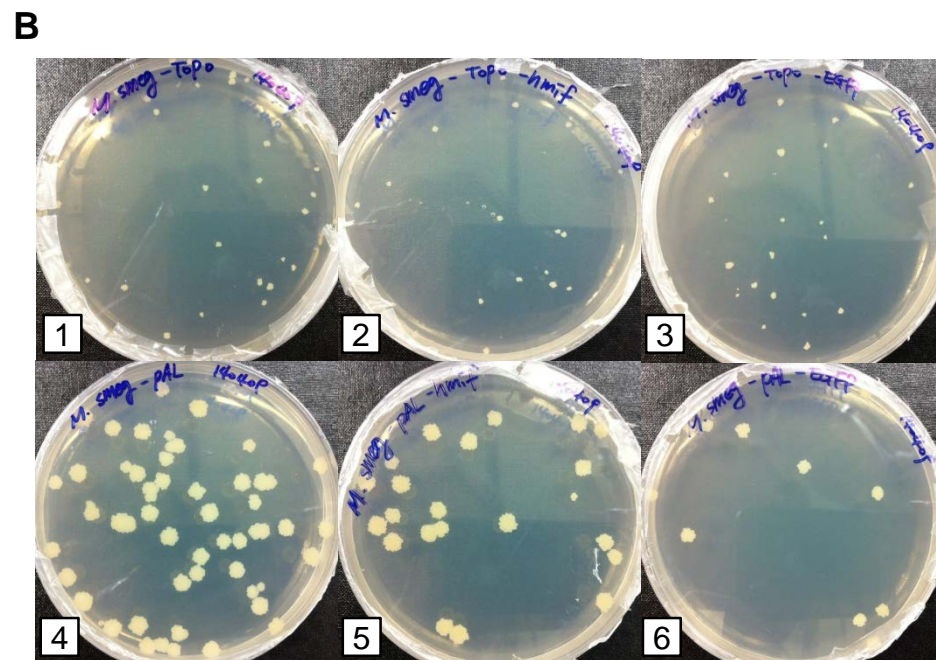

Supplement: S5 Fig — Comparison of the phenotypic differences between rSmeg carrying the pMyong2 or pAL5000 vector system. Three days after transformation, the colonies were (A) counted, and (B) the colony sizes were examined. 1, M. smegmatis pMyong2-TOPO; 2, M. smegmatis pMyong2-hmif; 3, M. smegmatis pMyong2-EGFPh; 4, M. smegmatis pAL5000-TOPO; 5, M. smegmatis pAL5000-hmif; 6, M. smegmatis pAL5000-EGFPh (PDF) [file pone.0122897.s005.pdf]

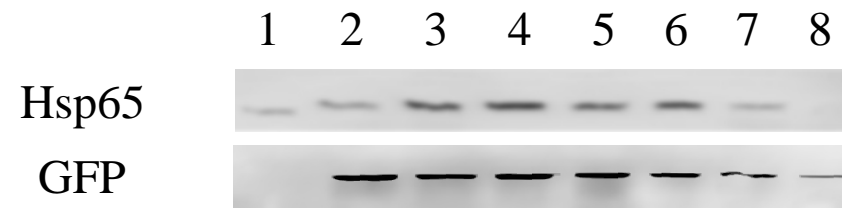

Supplement: S6 Fig — Protein extracted from M. smegmatis (lane 1), rSmeg clones carrying pMyong2-EGFPh for 10 generations (lane 2, 1st generation; lane 3, 2nd generation; lane 4, 4th generation; lane 5, 6th generation; lane 6, 8th generation; lane 7, 10th generation), and GFP protein (lane 8) as a positive control. Each generation time was three days. (PDF) [file pone.0122897.s006.pdf]
