# Supplementary material for: The Development of a Novel Mycobacterium-Escherichia coli Shuttle Vector System Using pMyong2, a Linear Plasmid from Mycobacterium yongonense DSM 45126T
Source: PLoS One. 2015 Mar 30;10(3):e0122897. doi: 10.1371/journal.pone.0122897 (PMC4378964; doi:10.1371/journal.pone.0122897)
Supplement: S2 Fig — (A) Predicted secondary structure of pMyong2 termini showing three hairpin structures. (B) The linear palindromic sequence is shown and the stem loop structures are indicated with different colors in panel A. (C) Alignment of the terminal sequences between pMyong2 and pCLP of M. celatum. (PDF) [file pone.0122897.s002.pdf]

A

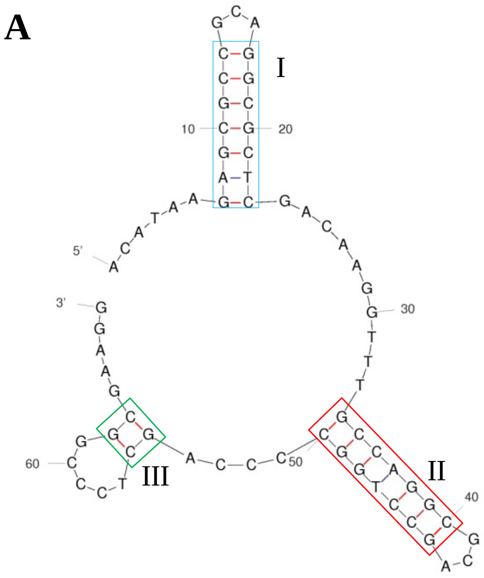

B

I II III

ACATAAGAGCGCCGCAGGCGCTCGACAAGGTTTGCCAGGCGCAGCCTGGCCCCAGCTCCCGGCGAAGG

C

```
pMyong2 (JQ657806)  A C A T A A G A G C G C C G C A G G C G C T C G A C A A G T T T G C C A G G C
pCLP(AF312688)      A G C A A A G A G C G C C G C A G G C G C G C G A C A G A T T T G C A G G G C

pMyong2 (JQ657806)  G C A G C C T G G C C C C A G C T C C C G G C G A A G - - - - - - - - - - - -
pCLP(AF312688)      G T A G C C C T G C C T C T C C A C C C G G C G A A G C C G G G G G G G G G G
```
