# Supplementary material for: The Development of a Novel Mycobacterium-Escherichia coli Shuttle Vector System Using pMyong2, a Linear Plasmid from Mycobacterium yongonense DSM 45126T
Source: PLoS One. 2015 Mar 30;10(3):e0122897. doi: 10.1371/journal.pone.0122897 (PMC4378964; doi:10.1371/journal.pone.0122897)
Supplement: S1 Table — (DOC) [file pone.0122897.s007.doc]

**Table S1. Primers used in this study**

| Primer name | Sequence of: | | Predicted |
| --- | --- | --- | --- |
|  | Forward primer (5’-3’) | Antisense primer (5’-3’) | size (bp) |
| pMyong2-ori | AAGCTTAGGCGGGCAACACGACATCTC | CCATGGGGGTTGTGGGCCGATGTGCT | 2469 |
| Hp | GGGGATATCAACGCGTCGGCGGCTGCGCGC | GCTCACCATGGTTGTGGCCATTGCGAAGTGATTCCTCCGGAT | 605 |
| EGFP | ATCCGGAGGAATCACTTCGCAATGGCCACAACCATGGTGAGC | CCCGATATCTTACTTGTACAGCTCGTCCA | 762 |
| pAL | GTGAGCCCACCAGCTCCGTAAGTT | ACGGATGCCACCACAGGCACT | 1893 |
| Hyg | TTAAGCTTGTCTGACAGTTACCAATGCTTAATCAG | AAGGATCCGGATGCCAGGGCCTTTCA | 1198 |
| Phsp65-mCherry | GGGGATATCGGTGACCACAACGACGCGC | AGGCGGCCGCTCAGTTATCTAGATCCGGTGGATCCC | 1175 |
| Phsp65-hmif | GGGGATATCGGTGACCACAACGACGCGC | AAGCGGCCGCTTAGGCGAAGGTGGAGTTGTTCC | 723 |
| OEM_p200020 | AACAACGCCCACCGCTGCTCAAA | GTGTTTGACGAGCTGACGAGTG | 220 |
| OEM_p200040 | GCTCGACACCACTCCTGCCCAACT | CTGATCGTGACGGACTGGATGACA | 565 |
| OEM_p200060 | CGCCTCGGCTCCCATTGTC | TGGTGGCCCGCAGACATTC | 649 |
| OEM_p200080 | ACGCGCTGGTAGTGCTCCCTTAG | CGAACACAAGCGCGACCACTACA | 284 |
| OEM_p200090 | CCTCGGCGGCGTAGTCAGTCA | GCCGGCCATATCACGATTCATTAC | 318 |
| OEM_p200100 | CTTGCTTTCGAGGTCTTTGA | GCGCCGCCGAGCAATACA | 603 |
| OEM_p200110 | CCGCCGGTATTGGGAAGG | GCACTGACAACACTCTGAA | 311 |
| OEM_p200120 | CCCGTGGCTGTGGAGACTTG | ACGGCTCAACGGGTGTCCTA | 519 |
| OEM_p200130 | GCGGTGCCACAGTGCCAGTAG | TCATGGACGAAGCCGACAGAGC | 342 |
| OEM_p200150 | ACACGGCGATCACGGGCTTAT | CGAGACACCATCCACCGAGAAAT | 219 |
| OEM_p200170 | CCGCTGCCGCACGAATACAT | CGTTGGCGGTCGATTCTTCACT | 498 |
| OEM_p200180 | GCGCAGCGGGCAATGGAG | ACACCCGCACCCCGTCTC | 285 |
| OEM_p200190 | TCCGAGGAAGACGAGTAGG | TCCGTCACAATCTGCCCCCTCACA | 215 |
| OEM_p200200 | GCGGAGATGGCATCCAC | GCTACGCCCCCTTCAAATA | 134 |
| OEM_p200220 | CGGGTGAGTCTTGGCGGCGGGGTA | ATCTCGTGCACGTAGAAGGAAA | 217 |
| OEM_p200230 | TTCTGTGTCGCCTATGCGGCCGGC | TCTGGCGATCGTGAAGACGAGCAC | 158 |
